# The parasite *Trichomonas vaginalis* expresses thousands of pseudogenes and long non-coding RNAs independently from functional neighbouring genes

**DOI:** 10.1186/1471-2164-15-906

**Published:** 2014-10-17

**Authors:** Christian Woehle, Gary Kusdian, Claudia Radine, Dan Graur, Giddy Landan, Sven B Gould

**Affiliations:** Institute of Molecular Evolution, Heinrich-Heine-University Düsseldorf, Düsseldorf, Germany; Department of Biology and Biochemistry, University of Houston, Houston, TX USA; Genomic Microbiology Group, Institute of Microbiology, Christian-Albrechts-University, Kiel, Germany

**Keywords:** *Trichomonas*, Non-coding RNA, Pseudogenes, Gene families, Genome Duplication, Stop codon suppression

## Abstract

**Background:**

The human pathogen *Trichomonas vaginalis* is a parabasalian flagellate that is estimated to infect 3% of the world’s population annually. With a 160 megabase genome and up to 60,000 genes residing in six chromosomes, the parasite has the largest genome among sequenced protists. Although it is thought that the genome size and unusual large coding capacity is owed to genome duplication events, the exact reason and its consequences are less well studied.

**Results:**

Among transcriptome data we found thousands of instances, in which reads mapped onto genomic loci not annotated as genes, some reaching up to several kilobases in length. At first sight these appear to represent long non-coding RNAs (lncRNAs), however, about half of these lncRNAs have significant sequence similarities to genomic loci annotated as protein-coding genes. This provides evidence for the transcription of hundreds of pseudogenes in the parasite. Conventional lncRNAs and pseudogenes are expressed in *Trichomonas* through their own transcription start sites and independently from flanking genes in *Trichomonas*. Expression of several representative lncRNAs was verified through reverse-transcriptase PCR in different *T. vaginalis* strains and case studies exclude the use of alternative start codons or stop codon suppression for the genes analysed.

**Conclusion:**

Our results demonstrate that *T. vaginalis* expresses thousands of intergenic loci, including numerous transcribed pseudogenes. In contrast to yeast these are expressed independently from neighbouring genes. Our results furthermore illustrate the effect genome duplication events can have on the transcriptome of a protist. The parasite’s genome is in a steady state of changing and we hypothesize that the numerous lncRNAs could offer a large pool for potential innovation from which novel proteins or regulatory RNA units could evolve.

**Electronic supplementary material:**

The online version of this article (doi:10.1186/1471-2164-15-906) contains supplementary material, which is available to authorized users.

## Background

The parabasalian flagellate *Trichomonas vaginalis* is a unique human parasite causing trichomoniasis, the most common sexually transmitted disease (STD) [1]. The anaerobic protist possesses the ability to rapidly shift between an amoeboid and flagellated phenotype [2, 3], and was once considered to represent an early-branching eukaryotic lineage [4]. At least 46,000 genes, and potentially up to 60,000, are encoded on six chromosomes, representing one of the highest coding capacities known [5, 6]. Exhaustive coding capacity analyses in *Trichomonas* are generally hampered through the extensive presence of repeats and transposable elements that are thought to make up 45% of the genome [7]. The expansion of the genome appears recent [5] and might coincide with the colonization of new host habitats. The genome enlargement of this eukaryote was further fueled by a high amount of lateral gene transfer events [5, 8] and the massive expansion of some gene families [9, 10]. It has been suggested that the frequency of pseudogenes in *T. vaginalis* is at least 5% and that unstable gene families that underwent many gene duplication events, thereby producing pseudogenes on the way, further contributed to the large genome of *T. vaginalis* [11].

The transcriptome of *T. vaginalis* and its many known strains is not well characterized, but some classes of non-coding RNAs (ncRNA) have been described. Genome annotations of *T. vaginalis* include 668 ribosomal RNAs (rRNA) genes of three types and 468 transfer RNAs (tRNA) genes of 48 types [5, 7]. RNA subunits of the ribonucleoproteins RNase P and MRP were also identified [12, 13]. Furthermore, small regulatory RNAs (sRNA) have been discovered including potential microRNAs (miRNA) [14–17], small nuclear RNAs (snRNA) [18] and small nucleolar RNAs (snoRNAs) [12, 14]. Genes of the Argonaute (AGO) and Dicer-like family are encoded by *Trichomonas* and hence suggest the existence of functional RNA interference mechanisms [5, 14], although other studies question the functionality of identified miRNAs in this parasite [19]. Regulatory RNAs are mostly small (<200 nucleotides), but recent reports of longer regulatory RNAs are accumulating [20–27]. Recent deep-sequencing of the parasite’s transcriptome has shed light on the expression potential of the genome and provided evidence for the expression of about 30,000 genes and a correlated co-expression of gene families induced by different stimuli [10, 28].

Long non-coding RNAs (lncRNAs) are often defined as transcribed but not translated RNA segments larger than sRNAs (>200 nucleotides) [29]. lncRNAs affect chromosomal dynamics, the telomeres and structural organization [20, 21, 23]. Their expression can be regulated and restricted to certain developmental stages and tissues [20, 22, 24]. Some are recognized by canonical transcription factors [30] and their promoters can show evidence of purifying selection [26]. However, the functionality of the majority of lncRNAs is unknown, and many are thought to represent “junk” RNA or transcriptional noise attributable to the promiscuity of RNA polymerase II [31]. It has been proposed that every euchromatic nucleotide in the human genome could be transcribed [32], albeit this does obviously not necessarily translate into every expressed nucleotide having a biological function [33]. Most lncRNA studies focus on metazoan organisms with yeasts representing a rare exception [25, 27, 34–36]. Although several thousand lncRNAs have been predicted to be functional [22, 25, 37], the number of experimentally validated functional lncRNA (about 200) remains low [38, 39]. Most lncRNAs contain only short open reading frames [39], but for yeast it has been demonstrated that more than a thousand short open reading frames are translated [40]. They were shown to be conserved between organisms and to fulfil biological functions [41–43].

Pseudogenes, like lncRNAs, do not encode functional proteins but can be identified through their sequence similarity to protein-coding genes from which they evolved. Some are expressed and translated, but most resemble non-processed genetic remnants [44–46]. There are 1354 annotated pseudogenes in *T. vaginalis* (or ~2% of predicted protein-coding genes), but based on gene family analysis it was estimated that a minimum of 5% of the protein-coding genes may represent pseudogenes and half of the *Trichomonas* transmembrane cyclase family appears to represent pseudogenes [11]. Expressed pseudogenes are essentially a sub-group of lncRNA, and for some a biological function has been identified [45, 47]. Antisense pseudogene transcripts can be processed into small regulatory RNAs [48, 49] or to complementarily bind to their functional counterparts and influence their expression [50, 51]. One of the best-studied functional lncRNAs that participates in X chromosome inactivation in mammals is the *Xist* RNA. It is a lncRNA that originates from the pseudogenization of a protein-coding gene [52].

Here we identified and characterized lncRNAs of the parabasalian parasite *T. vaginalis* by screening available transcriptional data and 271 million novel RNA-Seq reads we generated. We found that almost one fifth of the transcripts originate from intergenic regions of the parasite. We have characterized these transcripts in terms of their potential coding capacity, flanking genomic regions and similarity to annotated genes, in order to elucidate their origin and determine what drives their expression.

## Results and discussion

### General transcript mapping and homology

**Figure 1:**
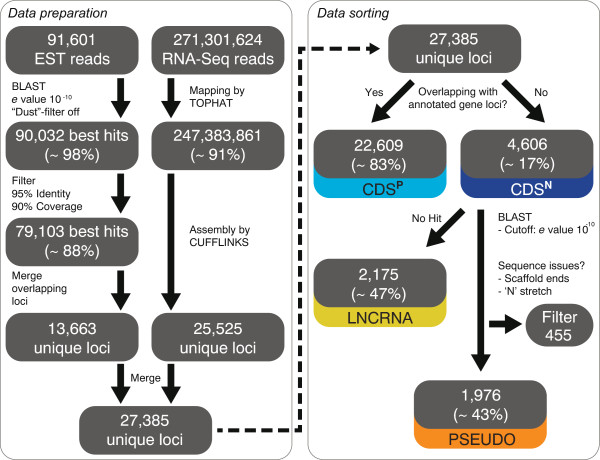
**Schematic workflow of the data management.** Sequenced reads and expressed sequence tags (ESTs) of *Trichomonas vaginalis* were mapped onto the genome as shown and sorted into the categories presented according to their best BLAST hits.

We used 91,601 expressed sequence tags (ESTs) downloaded from TrichDB [7] and combined those with 271.3 million raw reads from our own RNA-Seq data. After assembling and merging the two data sets, we mapped in total 27,385 unique transcript contigs onto the genome of *Trichomonas vaginalis* in total. From those, 22,609 (83%) mapped onto regions encoding annotated genes and 4,606 (17%) did not. We refer to these datasets as CDS^P^ and CDS^N^, respectively (Figure 1). The CDS^P^ set overlapped with 24,950 protein-coding genes, representing only 42% of annotated genes and less than half of what was found for other protists [53–56]. Yet, these transcripts represent 93% of the gene families identified in *Trichomonas* [57], indicating that (a) sequencing depth appears to be sufficient and that the numbers are not likely to change much with more sequencing data becoming available, and that (b) most of the functional proteome the genome encodes is expressed, but not all members of a gene family.

The homology of CDS^N^ transcripts to annotated genes was examined next. About half (2175; 47%) had no significant similarity to any annotated genes, hence representing lncRNAs of non-recognizable origin. The remainders of the CDS^N^ transcripts (2431; 53%) were found to be significantly similar to annotated genes and were thus classified as expressed pseudogenes with functional homologous genes. These were additionally filtered to exclude contigs that mapped to the very proximal regions of genomic scaffolds and those with bad sequencing resolution, that is stretches of ‘N’. 455 such contigs were identified. We termed the remaining identified set PSEUDO, and those loci without significant homologies LNCRNA (Figure 1).

The repetitive nature of this parasite’s genome is extensive. Using REPEATMASKER [58] we screened the genome for repetitive elements and subsequently for overlaps with associated genomic regions. About 30% of the PSEUDO and CDS^P^ loci (31.5% and 28.9%, respectively) were associated with repeat regions, while for the LNCRNA loci this was the case for only 17.3%. Comparable to PSEUDO and CDS^P^, a dataset consisting of all *T. vaginalis* gene annotations showed an association with repeat elements for 29.5%. Therefore, these loci seem to be preferably embedded into the repeat structure of the genome, but do not show any specific links. LNCRNA loci varied more and this might be connected to specific sequence selection to form functional RNA structures.

**Figure 2:**
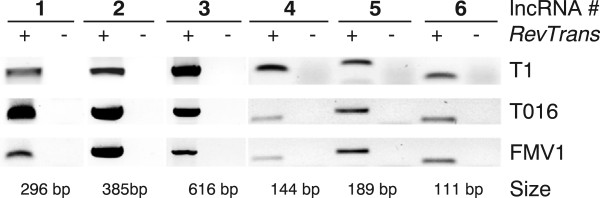
**Expression of lncRNAs is conserved among different** ***T. vaginalis*** **strains.** Reverse transcriptase (RT-)PCR was performed on complementary DNA (cDNA) generated from RNA of *T. vaginalis* strains T1, T016 and FMV1 in the presence (+) or, as control, absence of the reverse transcriptase enzyme (−). All six randomly chosen lncRNAs candidates were found expressed in the three different strains analysed.

Data for the human genome suggests that half of the transcriptome consists of lncRNAs [22] and in mouse 28,000 ncRNAs were identified [37]. For *T. vaginalis* only 17% of the transcripts did not map to any annotated genes. With more data for the parasite becoming available one will be able to determine whether this difference is due to sequencing depth or biological differences. Considering studies on other protists, which were able to cover most of the annotated genes with less sequencing depth, the former seems unlikely [53–56]. In any case, most will resemble transcriptional noise [31] and random expression caused for instance by sequences mimicking transcriptional promoters (see below), with only a few representing expressed and functional lncRNAs. We experimentally validated the expression of a random set of lncRNAs in the most frequently used laboratory strain T1, and the virulent T016 and highly virulent FMV1 strains. For all six cases we could verify expression in all the three *T. vaginalis* strains tested (Figure 2), which demonstrates lncRNA expression to generally be conserved across the different strains tested.

### Characterization of transcribed pseudogenes

The PSEUDO set includes 7% of all transcripts analysed. It represents a lower bound on the pseudogene content of *T. vaginalis*, as this set does not include non-expressed pseudogenes, unitary pseudogenes, or pseudogenes erroneously annotated as functional genes. It has previously been estimated that at least 5% of the annotated genes of *T. vaginalis* could represent mis-annotated pseudogenes, and for one large gene family it has been shown that about half of its members could qualify as pseudogenes [59]. For the human genome it is estimated that 8 to 20% of all pseudogenes are expressed [44, 46]. If that is also true for *Trichomonas*, the parasite could potentially harbour between 10,000 and 25,000 pseudogenes. In order to estimate the number of non-expressed pseudogenes in *T. vaginalis* we performed BLASTN searches (*e* value cutoff 10^−10^) with annotated proteins to intergenic regions lacking expression evidence. This revealed approximately 50,000 intergenic loci, for which no expression evidence exists, but with a significant homology to annotated (and likely functional) genes. Although the absolute number is much higher, the value is comparable to that from human, where the amount of pseudogenes (up to 20,000) almost reaches that for the coding genes [47]. High abundances of pseudogenes are generally known for mammals, but their number in less complex organisms is usually smaller [60, 61]. This would support a recent hypothesis that the *Trichomonas* genome (and maybe even proteome) faces constantly emerging and disappearing paralogs, and is in a steady state of changing [11].

Large gene families contain high a number of genes, where each one can pseudogenize or duplicate. We examined our transcribed and non-transcribed intergenic pseudogenes for a correlation between the number of pseudogenes and sizes of corresponding gene families. Although we observed a moderate Pearson correlation for non-transcribed pseudogenes (r = 0.54, *P* value <0.05), the correlation for transcribed pseudogenes (PSEUDO) was rather low (r = 0.19, *P* value <0.05), indicating a potential connection. But at least for the transcription of pseudogenes this factor seems less important. Functional categories of pseudogene datasets were analysed using EuKaryotic Orthologous Groups (KOGs; [62]) and it revealed similar distributions of categories for non-transcribed pseudogenes, transcribed pseudogenes (PSEUDO) and annotated transcripts (CDS^P^). A clear difference occurred according to the frequency of genes, which were associated with KOG categories. While for CDS^P^ 64% of loci remained unclassified, for the untranscribed pseudogenes and PSEUDO loci they accounted for 83% and 92%, respectively. 4% of unclassified loci in PSEUDO, which is low compared to 37% for non-transcribed pseudogenes, represented repetitive gene models described in Carlton et al. [5]. These findings indicate that these pseudogenes, which are still transcribed, predominantly are based on recent *Trichomonas*-specific functions.

**Figure 3:**
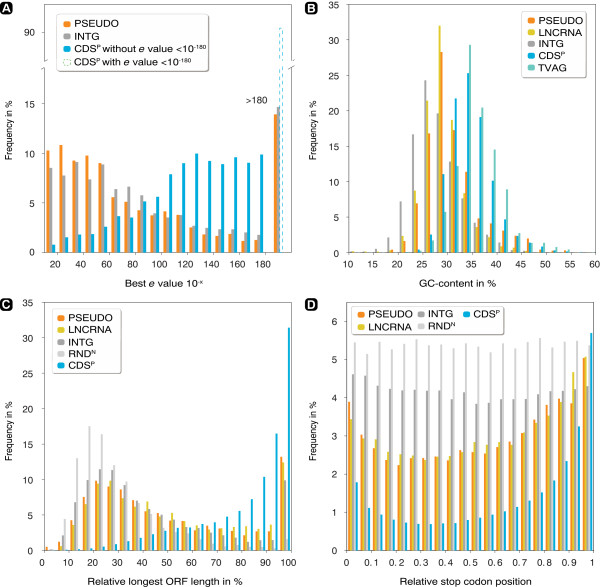
**Comparison of potential coding capacities for the different sets of transcripts identified. (A)** shows proportions of BLASTN hits with a given *e* value to annotated genes of *Trichomonas vaginalis*. Relative frequencies of CDS^P^ were calculated excluding those *e* values lower 10^−180^ (the dashed bar illustrates the relation compared to all CDS^P^ hits). **(B)** Distribution of the GC-contents in per cent, showing that CDS^P^ behaves nearly identical to TVAG (TVAG representing annotated genes of the parasite). **(C)** Distribution of the sequence lengths of the longest ORFs relative to the corresponding full-length sequence. The ORFs of CDS^P^ distribute very differently in comparison to the remaining datasets, while the intergenic regions behave similar to the PSEUDO and LNCRNA sets. **(D)** Distribution of stop codons over the relative positions in the full sequence of the reading frame showing the lowest number of stop codons. Counts were normalized according to total codons per bin.

In order to compare homologies of PSEUDO, CDS^P^ and intergenic regions (INTG; randomly picked intergenic loci, but with the same length distribution as the CDS^N^) we examined the distributions of the best BLASTN hit *e* values (Figure 3A). All compared sets differed significantly (Kolmogorov-Smirnov test; *P* value <0.05; Additional file 1: Table S1), with the INTG behaving similarly to the CDS^N^ set. The BLASTN hits of the PSEUDO set revealed higher *e* values compared to those of the CDS^P^ set, suggesting these homologies are less conserved and to only partially map onto the annotated gene sequences. The several cases of pseudogenes that retrieved hits with small *e* values – indicating full sequence hits – most likely represent novel pseudogenes that represent more recent gene duplications events and not falsely annotated genes.

### Transcript coding capacity of CDS^N^

The PSEUDO, LNCRNA and CDS^P^ sets were compared in regard to their potential protein-coding capacities. Three control sets were used: the first represents the intergenic loci (INTG) mentioned above, the second was based on randomized CDS^N^ sequences (RND^N^) and the third simply comprised all annotated *T. vaginalis* genes that included also those lacking expression evidence (TVAG; Table 1 and Figure 3B-D). We found that the PSEUDO and LNCRNA sets behaved similarly and were placed in between the protein-coding CDS^P^ and the randomized CDS^N^ sets. Differences between all datasets, except PSEUDO and LNCRNA in Figure 3D, were statistically supported (Kolmogorov-Smirnov test; *P* value <0.05; Additional file 1: Table S1), where the *P* values suggested that CDS^P^ differs the most. As expected for CDS^P^, this set’s GC-content was found to be very similar to the GC-content described for annotated genes (34.6% versus 35%, respectively), while the GC-content of CDS^N^ (30.5%) was more similar to that of the non-expressed intergenic sequences (28.8%). PSEUDO and LNCRNA subsets of CDS^N^ alone differ only slightly from the total CDS^N^ set, with the PSEUDO set showing a marginal tendency towards protein-coding gene sequences (Table 1). This suggests that the PSEUDO set does not contain many, if any, genes that are not yet annotated.

**Table 1:** **Protein coding sequence features of the various sets analysed**

Category			CDS^N^			
	TVAG^(1)^	CDS^P^	PSEUDO	LNCRNA	INTG^(2)^	RND^N(3)^
**Number**	59672	22609	1976	2175	4606	4606
**Median longest ORF length**	636	1002	195	165	156	120
**Mean longest ORF length**	917.64	1320.23	286.64	262.63	199.45	127.05
**Median relative longest ORF**	99.58%	89.19%	42.11%	44.69%	34.31%	24.52%
**Longest ORF ≥50 aa**	99.59%	98.92%	64.83%	55.82%	53.58%	26.90%
**Proportion of stop codons** ^**(4)**^	0.29%	1.45%	3.02%	3.08%	4.16%	5.38%
**GC-Content**	35.49%	34.62%	31.07%	29.42%	27.82%	30.52%

^(1)^Annotated protein-coding genes.

^(2)^Intergenic regions without expression evidence randomly selected in size of CDS^N^.

^(3)^Order of nucleotides randomized per sequence.

^(4)^In reading frame with lowest number of stop codons.

The relatively high amount of lncRNAs with longer open reading frames (ORFs; 55-65% ≥50 amino acids) is noteworthy. Similarities of lncRNAs to protein-coding genes have been described before and a high density of ORFs among lncRNA noticed [26, 39]. We found a median ORF length of 177 nucleotides among the CDS^N^ set, which is lower than the median of 250 nucleotides reported for mammalian lncRNAs [39]. As expected the PSEUDO and LNCRNA sets showed a significantly lower coding capacity when compared to the CDS^P^ set. It demonstrates that CDS^N^ does not just represent erroneous protein-coding gene annotations, but largely non-coding transcripts similar to the non-expressed intergenic regions.

**Figure 4:**
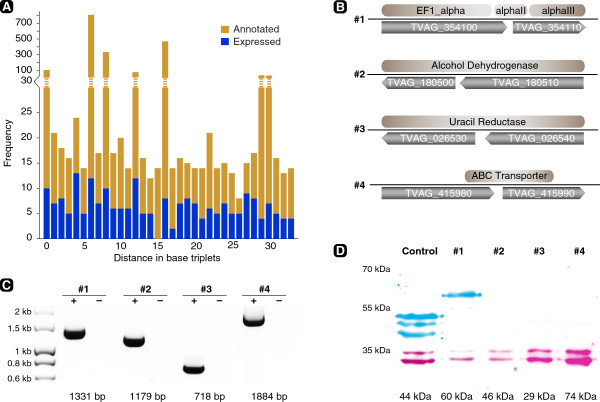
**No evidence for stop codon suppression in** ***Trichomonas vaginalis*** **. (A)** Bar diagram of the frequency of annotated gene pairs and their distances in base triplets (light grey). Dark grey bars indicate the gene pairs, for which expression evidence exists. Note that the most abundant distances originate from highly conserved and large gene families. **(B)** Illustration of four selected candidates, in which two adjacent genes share the same reading frame and in combination match to a single BLAST hit. **(C)** RT-PCR demonstrates the full-length transcription of the gene pairs including the C-terminal HA-tag. RNA was isolated from transfected trichomonads, transcribed into complementary DNA and served as template for the PCR using specific forward and HA-reverse primers (+). RNA served as a negative control (−). **(D)** Multiplex western blot analysis of the same candidates demonstrates only candidate #1 is translated. 50 μg of protein extract loaded, anti-HA in blue, anti-SCS (succinyl-coenzyme A synthetase subunit alpha; TVAG_047890; 33 kDa) in pink as a loading control, and TVAG_337240::HA served as a positive control. For SCS the double bands are routinely observed [64], and the two additional bands migrating below the 44 kDa TVAG_337240::HA fusion protein likely represent degradation products.

Cui and colleagues [59] suggested stop codon read-through could explain the high number of pseudogenes in *T. vaginalis*, and which are nearly identical to their evolutionary predecessors and functional counterparts. In consequence, a massive number of genes could have been missed during genome annotation. For a single candidate of the ABC transporter family, tentative evidence exists for stop codon suppression to occur in *Trichomonas* [63]. However, Western blot evidence for the translation of the full-length protein including its hemagglutinin (HA)-tag was not shown and the authors concluded: “*…further experimental work would be required to substantiate this*”. In the current *T. vaginalis* genome annotation we found 2,293 cases, in which two annotated genes on the same strand are separated by a maximum of up to 33 codons (Figure 4A; promoter and terminator sequences in the parasite are generally short, hence 99 nucleotides were chosen as an arbitrary cut-off value). For 219 of the 2,293 cases we found expression evidence existing across their combined length. These could represent misannotations, expressed pseudogenes, or cases of stop codon suppression leading to non-interrupted translation.

We selected four candidate loci (Figure 4B) and fused the two adjacent genes to a C-terminal HA-tag and checked for the transcription and translation of the fusion constructs in transfected cells. For one case (TVAG_354100 and TVAG_354110; together encoding the full-length elongation factor 1α) the mRNA reads we obtained and mapped, and our PCR amplification product, suggested an error in the genome assembly and an incorrect annotation (or a strain-specific difference), as the stop codon annotated between the two genes could not be verified. This construct served as an additional control next to the expression of TVAG_386160::HA. In all cases tested we found evidence for the expression of the full-length constructs, but not for their translation (Figure 4C-D). Only the control and the TVAG_354100::TVAG_354110 construct were translated and detectable through the C-terminal HA-tag. Alternative start codons do not appear to be used by the parasite either (Additional file 2: Figure S1A) and although the TAA stop codon is the most frequently encoded (64%), the other two, as expected, are functional (Additional file 2: Figure S1B). Hence, in summary, our results confirm a conservative codon usage by the parasite and that should stop codon suppression exist, it must be very rare and has yet to be experimentally verified.

### Distribution of CDS^N^ relative to flanking genes

For yeast it has been reported that the expression of lncRNAs is associated with the expression of functional genes encoded in flanking regions [65, 66]. We analysed the expression of the PSEUDO and LNCRNA sets of *Trichomonas vaginalis* depending on the four possible orientations to neighbouring genes: divergent (←CDS^N^→), convergent (→CDS^N^←), co-oriented (→CDS^N^→) and anti-oriented (←CDS^N^←). Distances and distributions of the orientations between PSEUDO and LNCRNA did show differences (see Table 2). The distance between PSEUDO loci and flanking genes was found to be larger compared to the LNCRNA set, while the LNCRNA loci were found in divergent orientations more frequently than a convergent one. Expression of PSEUDO and LNCRNA together with flanking genes in close proximity could indicate co-expression or even the expression as one RNA molecule. To statistically test the association of co-expression with upstream or downstream gens, we performed Yates’ chi-squared tests (Additional file 3: Table S2). All of the orientations tested, both for PSEUDO and LNCRNA, did not pass the false discovery rate (FDR; *P* value <0.05; Table 2), demonstrating that no statistically significant correlation regarding the expression of these sets together with their flanking genes.

**Table 2:** **PSEUDO and LNCRNA sets are expressed with no statistic significance in correspondence to flanking genes**

			Frequency		Mean distance (bp)		Statistics	
Dataset	Orientation		Absolute	%	Upstream	Downstream	***P***value	FDR
**PSEUDO**	Convergent		265	24.6	1419.4	1665.3	0.29	0.29
	Divergent		260	24.2	1485.3	1543.3	0.21	0.29
	Co-oriented		295	27.4	1286.8	1511.5	0.22	0.29
	Anti-oriented		256	23.8	1459.9	1508.0	0.03	0.10
**LNCRNA**	Convergent		233	17.5	1266.9	1207.4	0.42	0.55
	Divergent		434	32.6	1250.0	1162.6	0.13	0.34
	Co-oriented		329	24.7	1145.1	1283.7	0.69	0.69
	Anti-oriented		334	25.1	1430.1	1106.4	017	0.34

The mean intergenic distance between annotated genes in *T. vaginalis* was found to be 1165.4 nucleotides [5]. The mean distances to neighbouring genes for PSEUDO and LNCRNA range between 1100 and 1700 nucleotides (Table 2), being quite similar to that of the annotated genes. Overall the CDS^N^, PSEUDO and LNCRNA sets behaved “autonomously” and appear independently scattered when compared to flanking, annotated gene orientation and distance. Taken together this indicates that these transcripts are expressed independently from their neighbouring functional genes.

### PSEUDO and LNCRNA are transcribed, but lack obvious translation start motifs

**Figure 5:**
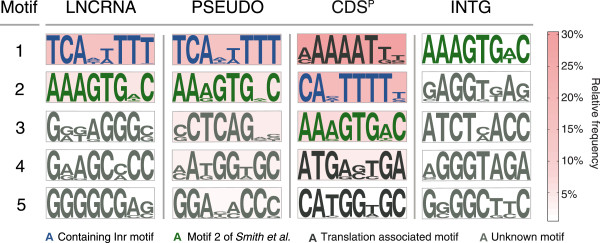
**Promoter sequence distribution.** Shown are pictograms and scores for the five best motifs (sorted by motif abundances) of the PSEUDO, LNCRNA, CDS^P^ and INTG sets. Background colour gradient indicates the frequency with which the motifs were identified. Note that the Inr motif of the CDS^P^ set misses the initial ‘T’; manual inspection revealed that 64% did however encode it. Translation initiation motifs containing an ‘ATG’ are only found among the CDS^P^ set.

Several promoter motifs including the DNA initiator motif (Inr) have been identified in *T. vaginalis* [67], and some are linked to the expression of gene subsets induced through changing environmental conditions [10]. In order to identify known, as well as new, promoter sequences, the upstream regions of the expressed intergenic loci were screened for overrepresented motifs (Figure 5). A motif similar to the Inr motif of the CDS^P^ (that is annotated and expressed protein-encoding genes) was well represented among upstream sequences of all expressed loci (PSEUDO, LNCRNA). With 16.8% for LNCRNA and 15.5% for PSEUDO, the frequency of the most prominent Inr motif was comparable to the 19.9% of the CDS^P^ set (Additional file 4: Figure S2). Among all loci we identified one non-functional pattern recently described as the M2 motif (AAAGTGAC) [67], but only among the CDS^P^ set the translation-associated M4 motif (AAAAT[T/G]) was identified together with other translation start motifs containing ATG start codons (Figure 5). PSEUDO and LNCRNA display approximately the same amount of known transcription-associated motifs, while lacking any evidence for translation-associated motifs. INTG sequences, for which we found no expression evidence, do not encode any of the previously described motifs, except M2, but with very low frequency.

Taken together this demonstrates that lncRNAs and pseudogenes in the parabasalian parasite are not expressed as by-products and in dependence to neighbouring genes as found for other model organisms [66], but because of their own transcriptional initiator motifs. As suggested by Carvunis and colleagues [40], and supported by our data, it is possible that the LNCRNA loci only represent an intermediate and transient form of genetic elements with characteristics from both functional proteins and intergenic regions. In either case, they would not simply represent transcriptional noise, but could serve as a sequence pool for the development of novel functional genes. This would further explain the high number of ORFs identified among the loci and the presence of fully functional promoter motifs. However, it is too early to tell whether any of these fulfil an actual biological function.

## Conclusion

The vast majority of information available on lncRNA stems from mammals [38]. No analysis dedicated to the characterization of lncRNA or pseudogene expression in protists apart from yeast [27, 35] is currently available. Our results provide insight into the expression of lncRNAs of a representative of the not well-studied eukaryotic kingdom of excavates. The expression of lncRNAs and pseudogenes in the parabasalian parasite *Trichomonas vaginalis* is extensive. Almost one-fifth of the transcripts mapped onto non-coding genomic loci, and of which half showed no sequence similarity to annotated genes of the protist. These loci do not encode for canonical proteins, but are clearly distinct from the random sequences that were simultaneously analysed as controls. Intriguingly, and in contrast to yeast [65], the expression of intergenic DNA is not associated with annotated neighbouring genes, but driven by transcription start signals mimicking those of coding genes. The fact that half of the lncRNAs expressed are pseudogenes reflects the dynamic nature of the *Trichomonas* genome that is characterized by an unknown amount of duplications of at least parts of the genome and large gene families that are unusually frequent.

## Methods

### Culture, RNA Isolation and cDNA synthesis

*Trichomonas vaginalis* strains T1, T016 and FMV1 were cultivated in tryptone-yeast extract maltose-medium (2.22% (w/v) tryptose, 1.11% (w/v) yeast extract, 15 mM maltose, 9.16 mM L-cysteine, 1.25 mM L(+)ascorbic acid, 0.77 mM KH_2_PO_4_, 3.86 mM K_2_HPO_4_, 10% (v/v) horse serum, 0.71% (v/v) iron solution (=1% (w/v) Fe(NH_4_)2(SO_4_)× 6H_2_O, 0.1% (w/v) 5-sulfosalicylacid)) at 37°C in Falcon tubes. To prevent bacterial contamination a penicillin/streptomycin mix was added to a final concentration of 100 μg/ml to media. Approximately 2.5×10^8^ cells were pelletized at 1,000× g for 10 min at 8°C and total RNA isolated using TRIzol® (Invitrogen) according to the manufacturer’s protocol. RNA was additionally digested with DNase (DNase I, RNase-free, Therma Scientific). 1 μg of DNase digested RNA was transcribed into cDNA using the “SuperScript III First-Strand Synthesis System for RT-PCR Kit” (Invitrogen) with specific primers as stated below or the iScript Select cDNA Synthesis Kit (Bio-Rad) using its random primer mix according to manufacturer’s protocol. The synthesized cDNA was used as template for test-PCRs using specific primers (Additional file 5: Table S3). Amplification products were sequenced for verification.

### Sequencing, mapping and assembly

RNA-Seq reads were produced by Illumina sequencing of *Trichomonas vaginalis* under different conditions (Infection and/or oxygen stress at several time points). *T. vaginalis* was cultured and RNA isolated as described in [10] and deep-sequencing was performed by Eurofins MWG (Ebersberg, Germany). Two sequencing approaches had been used: 100 basepairs paired-end reads. The filtered and trimmed reads used here are deposited in Sequence Read Archive (SRA) [68] under Accession SRA059159 (3′-library) and SRA129698 (paired-end reads).

Genomic scaffolds of *Trichomonas vaginalis*, sequences of annotated genes, genomic features (General Feature Format), orthologous gene clusters and additional EST sequences were downloaded from TrichDB V1.3 [7, 57]. KOG classifications were adopted from a previous study [10]. In order to determine repetitive elements in the genomic scaffolds REAPEATMASKER was used using default parameters, *Trichomonas vaginalis* as species definition and RMBLAST as the search engine. The reads of both RNA-Seq sequencings were mapped separately to the draft genome and the corresponding genome annotations of *Trichomonas vaginalis* using TOPHAT2 [69]. Assembly of overlapping reads was performed by CUFFLINKS [70] and the results of the two samples were merged by CUFFMERGE [70]. We supplemented the RNA-Seq with additional ESTs from TrichDB. ESTs were matched to the *T. vaginalis* scaffolds using BLASTN [71] with disabled filtering. Best BLASTN hits with an identity of at least 95% and query coverage of at least 90% were extracted, and overlapping hits were merged to unique loci and combined with overlapping loci from the RNA-Seq experiments using BEDTOOLS [72]. Transcribed loci on smaller scaffolds (<1000 nucleotides) were discarded due to missing gene annotations [5].

### Classification of transcribed loci

Gene entries downloaded from TrichDB were used to search for overlap between our transcribed loci and the gene annotations. Overlapping regions were classified as CDS^P^, while those remaining were referred to as CDS^N^. Additionally we created two datasets to serve as controls. For the intergenic dataset (INTG) we extracted all sequences longer than 1000 basepairs from the *T. vaginalis* scaffolds that were not annotated as genes (with a designated TVAG number), not identified through mapped transcripts (CDS^N^ and CDS^P^) and were not found in close proximity to the ends of scaffolds. From these we randomly sampled sequences of the same lengths as those in CDS^N^, thus ensuring an identical length distribution. As a second control set we subjected CDS^N^ sequences to a random permutation of nucleotide order (RND^N^). Homologies to annotated *T. vaginalis* genes were inferred by BLASTN searches of CDS^N^, CDS^P^ and INTG against the annotated gene sequences, with an *e* value cutoff of 10^−10^. CDS^N^ loci without hits were classified as LNCRNA. CDS^N^ loci with hits were removed, if either the hit or the query sequence included undetermined nucleotides (“N”) or was prematurely terminated due to scaffold termination. Remaining CDS^N^ loci were classified as PSEUDO. Estimates for non-expressed pseudogenes were produced by taking all BLAST hits of annotated genes to intergenic regions with an *e* value cutoff of 10^−10^ and merging those with overlapping locations into single entries. Resulting pseudogene loci were assigned to gene families and KOG categories based on their best BLAST hit to annotated genes with the mentioned *e* value cutoff.

Information on which strand transcribed loci are encoded was inferred by counting TOPHAT hits of the 3′-libraries that are overlapping with the corresponding gene locations. An orientation was assigned, if at least 90% of the matching hits lead to the same orientation. A control with CDS^P^ and the corresponding genes, for which orientations are known, revealed that for 86% of them a unique orientation was identified and 95.4% of them were congruent with overlapping annotations. For CDS^N^ we were able to assign orientations for 79% of the loci.

Protein-coding capacities were examined by two different methods. The length of the longest ORFs was defined as the longest peptide sequence in any reading frame beginning with the start of the sequence or a methionine and ending at the next stop codon or the end of the sequence. We defined the frequency of stop codons as the minimum count found inspecting all six reading frames separately.

### Flanking regions and stop codon read-through

For motif search upstream regions of transcribed loci were extracted −60 to 40 basepairs relative to the start position. Resulting sequences were clustered using CDHIT [73] with a cutoff of 90%. A search for the most overrepresented motifs was conducted using the MEME software V4.7 [74] with window size of 6–8 and zero or one occurrences per sequence. Orientations and distances of transcribed loci to surrounding annotated genes were extracted from genome annotations of scaffolds using their locations.

Candidates for stop codon read-through were determined by examining locations of genome features. We searched for gene pairs on the same strand with a distance from 0 to 33 full codons. Transcription of connected genes was determined by using CUFFLINKS results for the paired-end libraries only. Assembled transcripts had to span at least from the stop codon of the one gene to the start codon of the other.

### Cloning and transfection

All fragments were cloned into expression vector pTagvag2; for primer sequences refer to Additional file 5: Table S3. For lncRNA_ATG the artificial SCS promoter of pTagvag2 [75] was replaced by the putative, endogenous promoter region of the candidate (309 bp upstream of open reading frame). To check if all three classical stop codons are valid in *T. vaginalis*, we altered the stop codon of the HA-tag (TAA) into TGA and TAG and checked the length of the translation of the actin derivative TVAG_054030 (Additional file 2: Figure S1B). To identify potential stop codon suppression, pairs of adjacent genes, for which combined expression evidence was found based on our RNA-Seq data, fragments were amplified with the 5′ oligonucleotide binding to the start codon of first gene and the 3′ oligonucleotide replacing the stop codon of the adjacent gene with an HA-tag (Additional file 5: Table S3). All gene sequences were amplified using a proof-reading polymerase and verified through sequencing. 30 μg of the plasmid DNA was used for transfection of roughly 2.5×10^8^*T. vaginalis* cells using standard electroporation [76]. After four hours of incubation neomycine (G418) was added to a final concentration of 100 μg/ml for selection.

Protein samples were separated through standard SDS-PAGE and blotted onto nitrocellulose membrane. Membranes were blocked in 5% milk powder in Tris-buffered saline pH7 (blocking buffer) for 30 min. Blots were incubated with the primary antibodies at a dilution of 1:5,000 in blocking buffer either overnight (ON) at 4°C or for 1 h at room temperature (RT) and then washed 3× with TBS-T (TBS +0.1% Tween 20), followed by the incubation with the secondary, fluorescent antibodies (1:10,000) and identical subsequent washes in the dark. Fluorescence signal was detected using a ChemiDoc™ MP System (Bio-Rad). Antibodies used: monoclonal HA-antibody (Sigma H9658), antibody against succinyl CoA synthetase alpha subunit SCSα [64], Alexa fluor 488 donkey anti-rabbit and Alexa fluor 594 donkey anti-mouse antibodies (Invitrogen).

## Electronic supplementary material

Additional file 1: Table S1: Kolmogorov–Smirnov test *P* values of datasets in Figure 3. (PDF 168 KB)

Additional file 2: Figure S1: Expression and Western blot analysis of lncRNA_ATG and stop codon analysis (A1) Illustration of lncRNA_ATG consisting out of start codon followed by two stop codons and a putative open reading frame without an obvious start codon. LncRNA_ATG::HA is transcribed in two clones of transfected trichomonads shown by reverse transcriptase PCR and specific primers (A2), but not translated as shown by western analysis (A3). (B1) Illustration and Western (B2) of stop codon analysis on Actin (TVAG_054030,42 kDa). (PDF 526 KB)

Additional file 3: Table S2: Corresponding values for 2x2 Yates’ corrected X^2^ tests. (PDF 129 KB)

Additional file 4: Figure S2: Relative frequencies and *e* values of motifs shown in Figure 5. The background colors indicate relative frequencies in the corresponding datasets. (PDF 270 KB)

Additional file 5: Table S3: Primer used to validate lncRNA candidates. (XLS 22 KB)
