# Supplementary material for: The parasite Trichomonas vaginalis expresses thousands of pseudogenes and long non-coding RNAs independently from functional neighbouring genes
Source: BMC Genomics. 2014 Oct 17;15(1):906. doi: 10.1186/1471-2164-15-906 (PMC4223856; doi:10.1186/1471-2164-15-906)
Supplement: Supplementary file 1 — Additional file 1: Table S1: Kolmogorov–Smirnov test P values of datasets in Figure 3. (PDF 168 KB) [file 12864_2014_6630_MOESM1_ESM.pdf]

Figure 2A	Dataset	PSEUDO	INTG	CDS^P		
	PSEUDO	1	2.45E-07	0		
	INTG	2.45E-07	1	0		
	CDS^P	0	0	1		
**Figure 2B**		**PSEUDO**	**LNCRNA**	**INTG**	**CDS^P**	**TVAG**
	PSEUDO	1	8.36E-16	5.46E-79	1.99E-264	0
	LNCRNA	8.36E-16	1	1.06E-44	0	0
	INTG	5.46E-79	1.06E-44	1	0	0
	CDS^P	1.99E-264	0	0	1	0
	TVAG	0	0	0	0	1
**Figure 2C**		**PSEUDO**	**LNCRNA**	**INTG**	**RND^N**	**CDS^P**
	PSEUDO	1	0.02	2.73E-15	8.22E-129	0
	LNCRNA	0.02	1	8.77E-29	2.14E-174	0
	INTG	2.73E-15	8.77E-29	1	1.55E-102	0
	RND^N	8.22E-129	2.14E-174	1.55E-102	1	0
	CDS^P	0	0	0	0	1
**Figure 2D**		**PSEUDO**	**LNCRNA**	**INTG**	**RND^N**	**CDS^P**
	PSEUDO	1	0.26	1.66E-61	1.29E-61	0
	LNCRNA	0.26	1	6.76E-56	1.58E-56	1.52E-289
	INTG	1.66E-61	6.76E-56	1	2.04E-05	0
	RND^N	1.29E-61	1.58E-56	2.04E-05	1	0
	CDS^P	0	1.52E-289	0	0	1
