# Supplementary figures and images for: The parasite Trichomonas vaginalis expresses thousands of pseudogenes and long non-coding RNAs independently from functional neighbouring genes
Source: BMC Genomics. 2014 Oct 17;15(1):906. doi: 10.1186/1471-2164-15-906 (PMC4223856; doi:10.1186/1471-2164-15-906)

A1

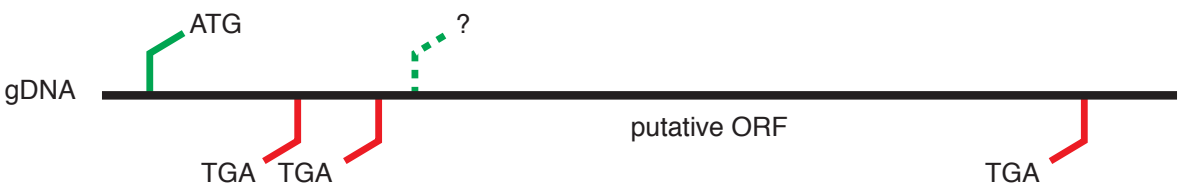

A2

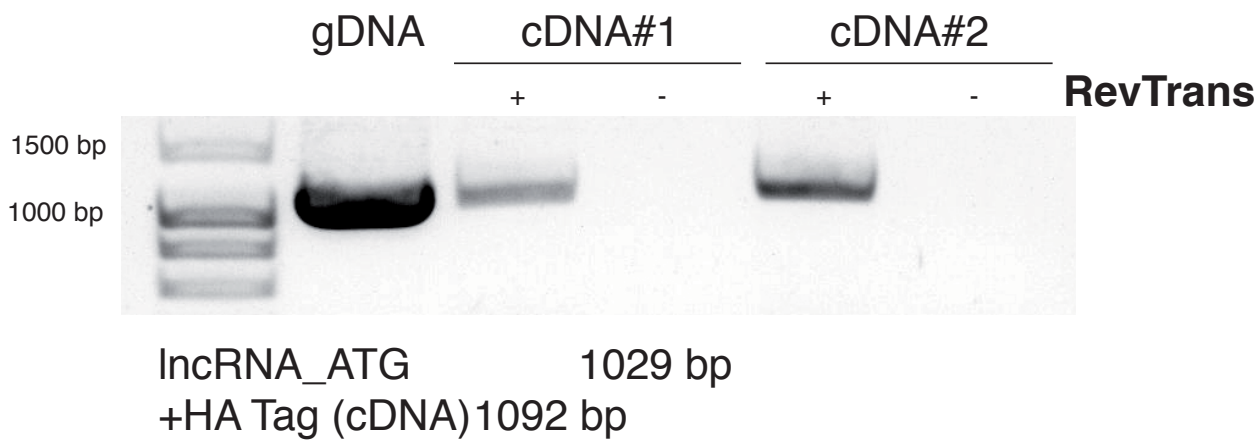

A3

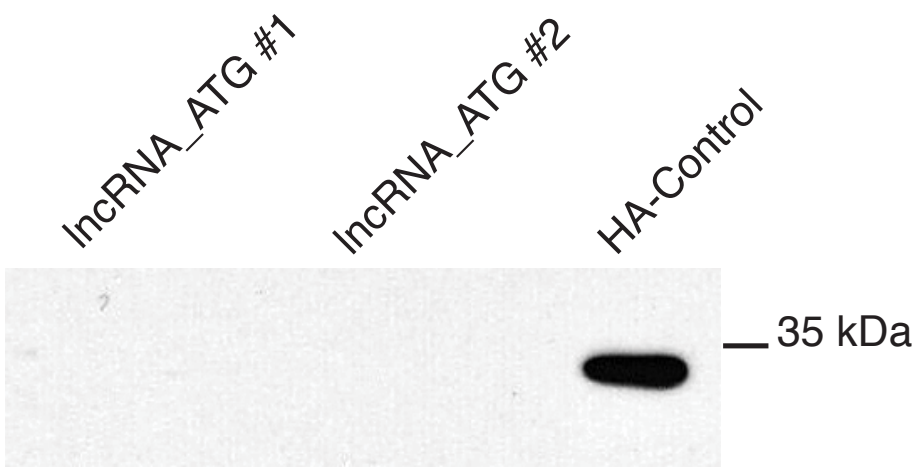

B1

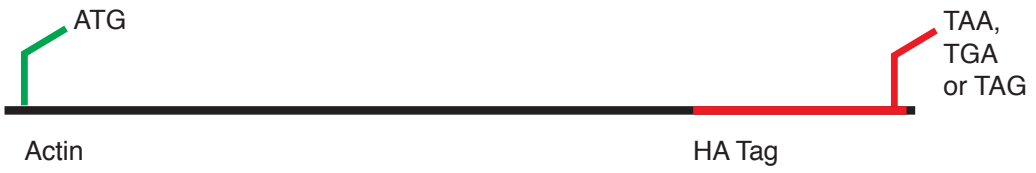

B2

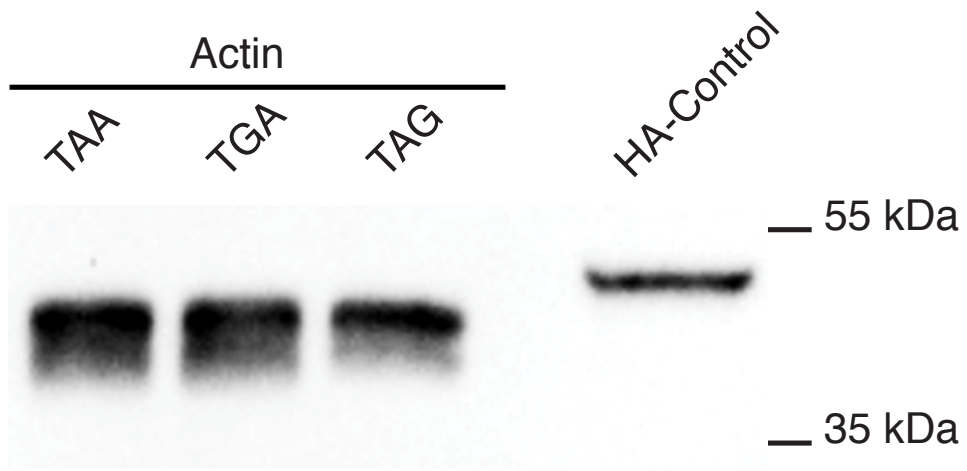

Supplement: Supplementary file 2 — Additional file 2: Figure S1: Expression and Western blot analysis of lncRNA_ATG and stop codon analysis (A1) Illustration of lncRNA_ATG consisting out of start codon followed by two stop codons and a putative open reading frame without an obvious start codon. LncRNA_ATG::HA is transcribed in two clones of transfected trichomonads shown by reverse transcriptase PCR and specific primers (A2), but not translated as shown by western analysis (A3). (B1) Illustration and Western (B2) of stop codon analysis on Actin (TVAG_054030,42 kDa). (PDF 526 KB) [file 12864_2014_6630_MOESM2_ESM.pdf]
