# Supplementary material for: The parasite Trichomonas vaginalis expresses thousands of pseudogenes and long non-coding RNAs independently from functional neighbouring genes
Source: BMC Genomics. 2014 Oct 17;15(1):906. doi: 10.1186/1471-2164-15-906 (PMC4223856; doi:10.1186/1471-2164-15-906)
Supplement: Supplementary file 3 — Additional file 3: Table S2: Corresponding values for 2x2 Yates’ corrected X2 tests. (PDF 129 KB) [file 12864_2014_6630_MOESM3_ESM.pdf]

Dataset	Orientation	Location	Transcribed	Non-transcribed
**PSEUDO**	Convergent	Upstream	214	51
		Downstream	203	62
	Divergent	Upstream	220	40
		Downstream	208	52
	Co-oriented	Upstream	241	54
		Downstream	228	67
	Anti-oriented	Upstream	203	53
		Downstream	180	76
**LNCRNA**	Convergent	Upstream	191	42
		Downstream	183	50
	Divergent	Upstream	377	57
		Downstream	360	74
	Co-oriented	Upstream	267	62
		Downstream	272	57
	Anti-oriented	Upstream	287	47
		Downstream	273	61
