# Supplementary material for: The parasite Trichomonas vaginalis expresses thousands of pseudogenes and long non-coding RNAs independently from functional neighbouring genes
Source: BMC Genomics. 2014 Oct 17;15(1):906. doi: 10.1186/1471-2164-15-906 (PMC4223856; doi:10.1186/1471-2164-15-906)

Motif	LNCRNA		PSEUDO		CDS^P		INTG	
	Sides	*e* value	Sides	*e* value	Sides	*e* value	Sides	*e* value
1	16.8%	1.0e-19	15.5%	8.8e-16	29.1%	2.3e-0611	2.7%	7.2e-43
2	5.5%	1.4e-49	5.7%	4.8e-22	19.9%	1.1e-1232	2.2%	1.0e-17
3	1.2%	3.7e-02	4.7%	1.7e-22	7.5%	7.4e-1156	1.6%	6.6e-18
4	0.9%	3.9e-01	3.2%	1.8e-11	4.0%	2.9e-0245	1.0%	6.9e-09
5	0.4%	8.0e+01	2.6%	2.9e-16	0.8%	3.7e-0075	0.6%	4.5e-06

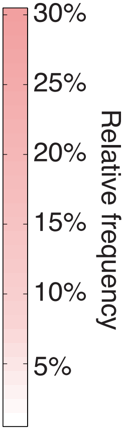

Supplement: Supplementary file 4 — Additional file 4: Figure S2: Relative frequencies and e values of motifs shown in Figure 5. The background colors indicate relative frequencies in the corresponding datasets. (PDF 270 KB) [file 12864_2014_6630_MOESM4_ESM.pdf]
